# Exploring the multifaceted roles of resuscitation-promoting factors in tuberculosis: Implications for diagnosis, vaccine development, and drug targeting

**DOI:** 10.1016/j.btre.2025.e00886

**Published:** 2025-03-13

**Authors:** Gamze Tanriver, Salman Ali Khan, Artur Góra, Novel N Chegou, Shima Mahmoudi

**Affiliations:** a Tunneling Group, Biotechnology Centre, Silesian University of Technology, Gliwice 44-100, Poland; b South African Medical Research Council Centre for Tuberculosis Research, Division of Immunology, Department of Biomedical Sciences, Faculty of Medicine and Health Sciences, Stellenbosch University, PO Box 241, Cape Town 8000, South Africa; c Biotechnology Centre, Silesian University of Technology, Krzywoustego 8, Gliwice 44-100, Poland

**Keywords:** Tuberculosis, Resuscitation-promoting factors (Rpfs), Diagnosis, Vaccine development, Drug target

## Abstract

Tuberculosis (TB) remains a significant global health challenge, necessitating continued research efforts to unravel its complex pathogenesis and advance diagnostic, therapeutic, and preventive strategies. Resuscitation-promoting factors (Rpfs) are peptidoglycan-hydrolyzing enzymes that have gained attention due to their key roles in TB infection dynamics. This review aims to provide a comprehensive overview of Rpfs in TB, highlighting their immunological roles, diagnostic potential, and implications for vaccine development and drug targeting through both in silico and experimental approaches. Rpfs exhibit diverse roles in TB, influencing bacterial resuscitation from dormancy, and immune modulation through interactions with host immune cells, such as dendritic cells, and they are potential targets for novel diagnostic and therapeutic interventions. Their ability to stimulate immune responses, particularly interferon-γ production by T cells, underscores their potential as vaccine candidates against TB. Moreover, Rpfs represent promising targets for drug discovery, with inhibitors potentially disrupting bacterial resuscitation and growth.

## Highlights

• Resuscitation-promoting factors (Rpfs) are critical enzymes that facilitate the resuscitation of *Mycobacterium tuberculosis* from dormancy, a key factor in TB persistence and latency.
• Rpfs interact with host immune cells and stimulate immune responses, positioning them as potential candidates for vaccine development.
• Due to their involvement in TB infection dynamics, Rpfs are promising targets for novel diagnostic tools.
• The design and development of Rpf inhibitors could prevent the reactivation of latent TB infection, thereby reducing the risk of disease progression and transmission.

## Graphical abstract

Rpfs play a crucial role in TB research by influencing key areas such as diagnostics, vaccine development, and therapeutic targeting.

**Figure:**
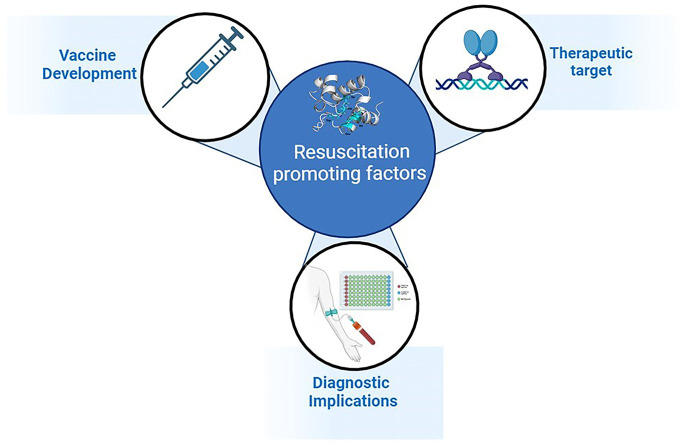

## 1 Introduction

Tuberculosis (TB) still remains a major global health challenge, with millions of new cases reported each year and substantial morbidity and mortality rates worldwide [1]. Our understanding of TB has evolved beyond the binary classification of active and latent infection, with current perspectives recognizing it as a spectrum ranging from latent TB infection (LTBI), incipient or subclinical stages, which may present with or without symptoms, to fully developed active disease [2,3].

While latent TB infection (LTBI) involves dormant *Mycobacterium tuberculosis* (*Mtb*) without clinical symptoms, active TB exhibits evident clinical symptoms and bacterial replication. Unfortunately, existing diagnostic tools often struggle to definitively differentiate between these states, leading to uncertainty in clinical decision-making. Addressing these gaps and limitations is crucial, underscoring the need for developing improved diagnostic tools capable of accurately predicting active TB disease. Specifically, new diagnostic tools are needed that not only identify individuals who are latently infected and at risk of developing active TB but also differentiate between various stages of TB infection, such as incipient or subclinical TB [2]. The immune response against *Mtb* involves multiple T cell subsets, making it essential to identify the antigens triggering T cell immune responses. This process is crucial for understanding protective immunity, immunopathology, and for developing novel vaccine candidates, diagnostics, and drug targets for TB [4]. By identifying *Mtb* antigens recognized by T cells, researchers have laid the groundwork for vaccine design, diagnostic assays, and personalized treatment strategies. This knowledge advances TB therapy and enhances clinical management, contributing to global TB control efforts [[5], [6], [7], [8]].

The diverse antigenic landscape of *Mtb* plays a pivotal role in shaping the immune response and the subsequent course of disease. Throughout various stages of infection, *Mtb* expresses a multitude of antigens, each eliciting distinct T cell responses and contributing to the overall functional heterogeneity of the immune system's defense against the pathogen [5].

Research has shown that individuals infected with *Mtb* who do not progress to active TB exhibit immune responses to a distinct set of *Mtb* antigens linked to latent infection. LTBI antigens, characteristic of slow-replicating *Mtb*, may be essential for future vaccines. Incorporating these antigens could activate immune responses that block the progression from *Mtb* infection to active TB [9,10].

Resuscitation-promoting factors (Rpfs) are a group of proteins with small molecular weight initially identified in *Micrococcus luteus*. These proteins play a crucial role in promoting the resuscitation of dormant bacilli, leading to the formation of normal, viable colony-forming bacteria [[11], [12], [13]].

The rpf genes in *Mtb* are differentially expressed across various stages of growth and under different physiological stress conditions, highlighting their specialized roles in bacterial survival and reactivation [14]. Under acidic conditions, which mimic the hostile environment inside macrophage phagosomes, rpfs show increased expression, likely aiding in bacterial persistence and survival. In contrast, hypoxic stress, which mimics the low-oxygen conditions within granulomas, induces higher expression of rpfs, facilitating bacterial adaptation to oxygen-limited environments and potentially priming the bacteria for future reactivation [15].

In this review, we aim to provide a comprehensive overview of the current understanding of Rpfs in *Mtb*, highlighting their potential as diagnostic markers, vaccine design, and drug targets from both experimental and computational perspectives.

## 2 Dormancy of *Mtb*

Dormancy in *Mtb* describes a state where the bacteria remain viable but exhibit minimal metabolic activity in response to unfavorable conditions and immune pressures within the host.

**Fig. 1:**
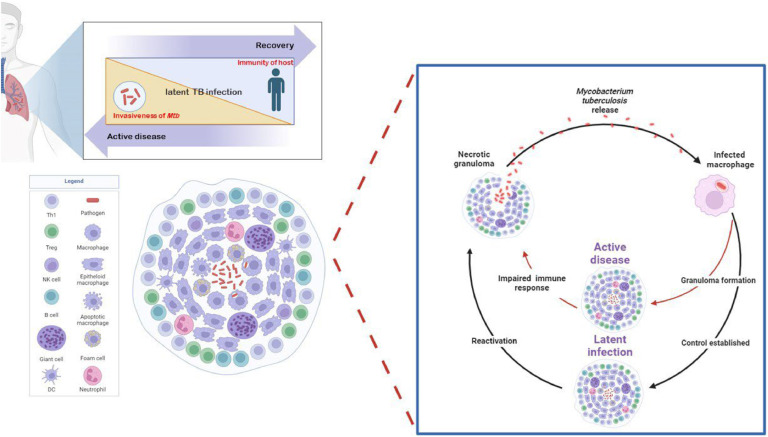
The interaction between host immunity and *Mtb* in maintaining LTBI. Created with BioRender.com. After host inhalation of *Mtb*-containing droplets, the bacteria are recognized and phagocytosed by resident alveolar macrophages. These macrophages secrete pro-inflammatory cytokines and chemokines in response to infection. Lung-resident DCs migrate to the trachea and alveoli to detect pathogens, then move to surrounding lymph nodes to present *Mtb* antigens to CD4^+^ T lymphocytes. Granulomas involve a complex immune response with multiple cell types including neutrophils, DCs, eosinophils, and mast cells, lymphocyte populations including macrophage, T cells, B cells, NK cells, and nonhematopoietic cells such as fibroblasts, endothelial and epithelial cells. LTBI represents a balance between the host's immunity and the invasiveness of *Mtb*. If the host's immune response is strong, the immune cells effectively eliminate the bacteria, and the person recovers. Conversely, if the immune system is weak, *Mtb* can multiply within the granulomas, eventually breaking through their containment and leading to active TB. When the host's immunity and the pathogen's invasiveness are balanced, the host remains latently infected.

LTBI is characterized by a dynamic equilibrium between the host's immune defenses and the invasive potential of *Mtb* (Fig. 1). If the immune response is strong, immune cells, such as macrophages and dendritic cells (DCs) can effectively eliminate the bacteria, leading to recovery. Conversely, a weak immune response allows *Mtb* to multiply within the granulomas, potentially breaching their containment and progressing to active TB. When the immune response and the pathogen's invasiveness are balanced, the bacteria remain contained, and the host stays latently infected without developing active disease [16].

The relationship between impaired immune responses and necrosis in TB granulomas is complex and sometimes controversial. While necrosis, characterized by cell death and caseous core formation, is often seen as immune failure, growing evidence suggests it may serve a dual purpose — both as a result of immune dysfunction and as a host mechanism to contain *Mtb* [17]. Granulomas play a crucial role in arresting the growth and proliferation of *Mtb*. Within these granulomas, *Mtb* is confronted with a challenging environment characterized by hypoxia, nutrient deprivation, and high nitric oxide (NO) concentrations. These conditions collectively create a hostile environment that limits the ability of *Mtb* to thrive and multiply, thus contributing to the containment of the infection [18].

The dormant *Mtb* exhibits several key features, though the factors contributing to latent and reactivation of TB remain poorly understood [[19], [20], [21]]. In the dormant state, replication is impossible, and the state is described as “viable but nonculturable” or “nongrowing but metabolically active,” due to the expression of numerous mycobacterial genes such as *relA*, which controls ATP and GTP syntheses, DNA replication, and protein synthesis. Expression of the dormancy regulon is sensitive to O_2_, NO, and CO and is ultimately controlled by the Dos three-component system (DosR/S/T). The Dos system includes two histidine sensor-kinases, DosS and DosT, and a regulatory element, DosR, which acts as a transcription factor when phosphorylated by the sensors [22]. DosS and DosT are membrane- tethered proteins containing a heme group and a histidine kinase, both crucial for autophosphorylation and subsequent signaling. DosT remains inactive when its heme is bound to O₂; however, as oxygen levels decrease (hypoxia), oxygen dissociates from the heme, triggering autophosphorylation. On the other hand, DosS remains inactive when the iron is in its ferric (Fe³⁺) state, but it becomes active and undergoes autophosphorylation when the iron is reduced to the ferrous (Fe²⁺) state [23]. The system is further regulated by gasotransmitters, like NO and CO, which stabilize the heme groups and maintain signaling. The NO-sensitive serine/threonine protein kinase, PknH, enhances DosR phosphorylation, crucial for full activation of the Dos regulon [24], which induces the transcription of about fifty coregulated genes that help *Mtb* adapt to hypoxic and anaerobic conditions, enabling its transition to a dormant, nonreplicating state aiding the transition to dormancy [21,25].

In addition, metabolic changes in *Mtb* inhibit cell processes and arrest the cell cycle, while increasing the synthesis of atypical enzymes like those in the glyoxylate pathway, allowing *Mtb* to utilize lipids as a carbon source [26]. Several redox-sensitive systems in *Mtb* are involved in both drug resistance and susceptibility [20]. Drug tolerance is acquired as dormant *Mtb* cells express ABC transporters, lack catalase-peroxidase necessary for isoniazid activation, and exhibit cell-wall thickening, contributing to antibiotic resistance [19,27,28].

## 3 Resuscitation-promoting factors in *Mtb*

Rpfs were the first mycobacterial proteins discovered to be linked with the reactivation of dormant cells [29,30]; however, the exact mechanisms by which *Mtb* bacilli persist in the dormant state and reactivate are not yet fully understood [21]. The Rpfs are found in Gram-positive organisms with high G + C content in their DNA, such as *Mtb* [31]. Rpfs are encoded by a cluster of five genes within the *Mtb* genome (*rpfA-Rv0867c, rpfB-Rv1009, rpfC-Rv1884c, rpfD-Rv2389c, rpfE-Rv2450c*) [29]. These proteins are pivotal in triggering the reactivation of dormant bacilli, a process crucial for the transition from LTBI to active TB [32,33].

Throughout different growth stages and under various stress conditions, rpf genes exhibit distinct expression patterns. During early resuscitation, all rpf genes are activated, with rpfA and rpfD showing the highest expression levels. Under nutrient starvation, rpfC maintains steady expression, while acid stress increases the expression of rpfD and rpfE. In response to hypoxia, both rpfC and rpfE expression is upregulated. These dynamic expression profiles suggest that different rpf genes play specialized roles in adapting to environmental stress and promoting bacterial survival [15,34].

Studies have underscored the antigenic nature of Rpfs, with their proteins stimulating the growth of non-replicating mycobacterial cells *in vitro*. Moreover, Rpfs play a significant role in the persistence and reactivation of chronic *Mtb* infection, particularly in animal models like mice [35,36]. Some preliminary findings suggest that Rpf supplementation could enhance the speed and sensitivity of *Mtb* culture [13,37,38]. The potential to reduce the time to positivity in culture-based *Mtb* tests by adding Rpfs presents an exciting advancement. Incorporating Rpfs into standard culture methods, either as recombinant proteins or as part of culture supernatant, offers a promising approach to speeding up the detection of *Mtb*. However, these initial results warrant validation through prospective clinical diagnostic studies.

## 4 Structural and functional characteristics of Rpfs

**Fig. 2:**
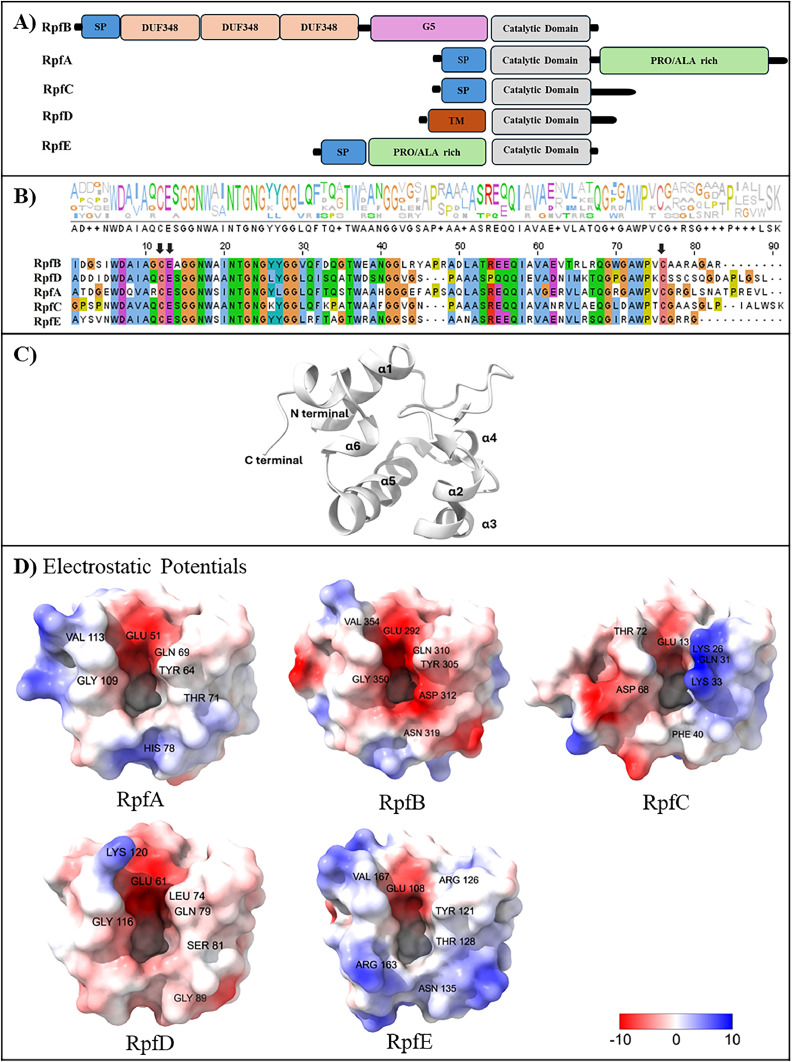
(A) Schematic domain compositions of the Rpf family. (SP: signal peptide and TM: transmembrane.) (B) The logo representation and multiple sequence alignment of the catalytic domain were generated using Jalview [100] and Clustal Omega programs [101], respectively. Arrows denote the conserved catalytic Glu and Cys residues belonging to disulfide bridge. The height of each residue in the logo reflects its conservation level, with taller letters representing higher conservation. Each amino acid is color-coded based on its chemical properties: polar (green), hydrophobic (blue), negative charge (magenta), positive charge (red), prolines (yellow), aromatic (cyan), cysteine (pink), glycine (orange), and unconserved (white). (C) Cartoon representation of RpfB (PDB ID: 4KL7 [44], chain A). (D) Electrostatic potential of catalytic domain of Rpfs (RpfA-AF3 model, RpfB (PDB ID: 4KL7 [44]), RpfC (PDB ID: 4OW1 [46]), RpfD-AF3 model, RpfE (PDB ID: 4CGE [48]). The figures were generated using ChimeraX (version 1.8) [102].

This section provides structural and functional insights on five known Rpfs (A–E) as mentioned above (Fig. 2). To date, some truncated apo structures of Rpfs (B, C, E) or RpfB in complex with triacetyl-beta-chitotriose (triNAG) and benzamidine (BEN) have been resolved, particularly catalytic domain using X-Ray or NMR spectroscopy; however, there are no available structures of RpfA and RpfD (Table S1). The Rpf family contributes to *Mtb* resuscitation by hydrolyzing peptidoglycan (PGN), the main component of the *Mtb* cell wall [13,39,40]. Structurally, all Rpfs share a conserved catalytic domain comprising approximately 70 amino acids, including six alpha helices, and adopt similar folds with each other and c-type lysosome [[41], [42], [43], [44], [45], [46], [47], [48]] (Figs. 2 A–C, S1A-B). This catalytic domain is characteristic of lytic transglycosylases [39] and exhibits lysozyme-like activity. The multiple sequence alignment including logo representation also points out conserved residues across all Rpfs catalytic domains (Fig. 2 B). The sequence identity of the catalytic domains is also in the range from 50 % to 74 % [44,46].

RpfA, which consists of 407 residues, contains both a catalytic domain and a Pro/Ala rich domain (Fig. 2 A). The catalytic domain includes the catalytic Glu51 and is characterized by a hydrophobic pocket formed by Val47, Trp56, Leu68, Phe70, Ile96, along with Trp111 and the disulfide bridge (Cys50-Cys114).

RpfB, consisting of 362 residues, is the most extensively studied and intricate member of the Rpf family [[41], [42], [43], [44], [45]]. Therefore, this review primarily focuses on the structural and functional characteristics of RpfB. It is notably unique in its role, as its deletion leads to delayed reactivation from chronic TB [49]. RpfB includes a signal peptide, a catalytic domain (80 residues), a G5 domain, and three domains of unknown function (DUF348 domains) (Fig. 2 A and C). The catalytic domain consists of Glu292 with its hydrophobic pocket including Ile288, Trp297, Val309, Phe311, Ile337, and Trp352 as well as a disulfide bridge (Cys291-Cys355). This hydrophobic pocket and Glu292 are conserved in other Rpfs, indicating the importance of this pocket for the catalytic glutamate and the catalytic mechanism of these enzymes. Furthermore, residues 325–327 of RpfB are found only in RpfA and are absent in other members of the Rpf [44] Regarding the G5 and DUF348 domains, they are not present in the other Rpfs. The G5 domain of RpfB is distinguished by a unique β-TH-β motif, with Gly229, Gly232, Gly245, and Gly269 and it is critical for its structural flexibility and cell wall adhesion [41]. This domain directly binds to Toll-like receptor 4 (TLR4), leading to the activation of dendritic cells and promoting Th1 T cell immunity [50]. The DUF348 domains exhibit a ubiquitin-like structure and possess a hydrophobic core with Val141, Leu143, Val159, and Leu163 residues. This hydrophobic core is essential for maintaining stability and facilitating interactions with other molecules [41]. Taken together, the catalytic domain hydrolyzes PGN, while the G5 and DUF348 domains contribute to PGN adhesion/binding [41,51,52]. In addition, the endopeptidase Rpf-interacting protein A (RipA) interacts with RpfB and RpfE, leading to synergistic hydrolysis of the bacterial cell wall by hydrolysis of peptidoglycan glycosidic and peptide bonds [[53], [54], [55]].

RpfC, consisting of 176 residues, includes the catalytic domain and an N-terminal signal sequence, which is presumed to have an extracellular role [46,47]. The catalytic domain includes Glu13 with a nearby hydrophobic pocket formed by Val9, Trp18, Leu30, Phe32, Ile55, and Trp70. Maione et al. suggest that a highly conserved disulfide bridge between Cys12 and Cys73 in RpfC may modulate the shape of the catalytic cleft of RpfC [47]. Mutations in these residues (one or both) can dramatically weaken its muralytic activity and inhibit bacterial resuscitation in *Mtb* [29,47].

Similar to other members of the family**,** RpfD (154 residues), has a catalytic domain, formed by Glu61 with Ile57, Trp66, Leu78, Ile80, Ile103, and Trp118 along with a disulfide bridge (Cys60-Cys124).

Lastly, RpfE (172 residues) consists of a catalytic domain and a Pro/Ala rich domain. It contains a conserved Glu16 with a hydrophobic pocket and disulfide bonds between Cys15 and Cys76 that are essential for its structural integrity and function, similar to other Rpfs [48]. Unlike RpfB, RpfE lacks the two short 3_10_-helices found in the α2–α3 loop. Additionally, the catalytic cleft of RpfE is wider than that of RpfB, resulting in a larger surface area and volume for the catalytic cleft.

In summary, the Rpf family exhibits a distinct lysozyme-like structure with a conserved catalytic glutamate, a hydrophobic pocket, and the disulfide bridge residues. These residues play essential roles in maintaining the protein's structural integrity and enabling effective interaction with peptidoglycan. The catalytic domain of the family is essential for bacterial cell wall metabolism, influencing both the degradation and remodeling of PGN, processes crucial for bacterial growth, division and resuscitation from dormancy [14,56]. The following section, titled ‘Drug targeting of Rpfs’ provides a detailed investigation of the catalytic domain, offering structural insight into its potential as a drug/inhibitor target.

The most remarkable difference among Rpfs stems from the electrostatic potential surface of the catalytic clefts (Fig. 2 D). Note that due to the lack of crystal structures for RpfA and RpfD, AlphaFold3 (AF3) [57] predicted structures are presented in Fig. 2 D. The catalytic domain of RpfE is basic (calculated pI = 9.5) when compared to the acidic catalytic domain of RpfB (calculated pI= 5.5) [48]. This charge difference may alter the interaction of the catalytic domain with modeled cross-links, that is, the interaction of isoglutamate with RpfE and isoglutamine with RpfB. These differences may imply differential functional roles of RpfE compared to other Rpfs [14,38]. Furthermore, two lysines (Lys26 and Lys33) in RpfC induce a different charge distribution compared to the rest [46]. Fig. S1C also presents different residues that can cause changes in charge distributions. Notably, the differences in catalytic site residues among Rpfs may lead to different charge distributions around the catalytic binding pocket which may play a role in specificity (substrate specificity) among them and alter interaction pattern [41,44,58]. Squeglia et al. also mentioned that negatively charged catalytic cleft can be linked to higher activity of RpfB, though this requires further investigation [44]. Mavrici et al. suggest that each Rpfs operates most effectively at specific pH levels or in hydrolyzing particular micro-domains of peptidoglycan [48].

Overall, the structural understanding of Rpfs is essential for understanding their role in TB pathogenesis and developing novel therapeutic strategies for latent TB infection and vaccine design. Due to the highly conserved catalytic residues, sequence identity and similar fold, the five Rpfs from *Mtb* can target similar substrates with similar binding orientation and catalyze hydrolysis by similar mechanisms [48]. However, variations in the electrostatic potential surfaces surrounding the catalytic cleft indicate functional specialization, potentially impacting substrate interactions and peptidoglycan hydrolytic activities (specificity towards different peptidoglycan modifications).

## 5 Diagnostic applications, vaccine development, and therapeutic targeting of Rpfs

Rpfs exhibit a broad range of clinical and biomedical significance. This section summarizes their role in TB, spanning from diagnosis implications and vaccine development, to therapeutic targeting.

### 5.1 Diagnostic implications of Rpfs in TB

Sequence analysis suggests that Rpfs are likely secreted by *Mtb* and possess extracytoplasmic functions. These characteristics make Rpfs potential targets for recognition by the host immune system, particularly during the reactivation to TB disease [59].

Their role in TB infection, affecting both cellular and humoral immune responses, highlights their potential as diagnostic markers. Previous studies have extensively investigated the immunogenicity of Rpfs, assessing both cytokines [35,[60], [61], [62], [63], [64], [65], [66], [67], [68], [69], [70], [71], [72], [73]] and antibody responses [59,74,75]. These investigations have provided valuable insights into the immune recognition of Rpfs and their potential as diagnostic markers for TB.

Current immunodiagnostic tests for *Mtb* infection focus on detecting the immune response to mycobacterial antigens [76]. Traditionally, the Tuberculin Skin Test (TST) has been widely used for diagnosing TB infection. However, TST has some limitations, primarily due to cross-reactivity with other nontuberculous mycobacterial infections and Bacillus Calmette-Guérin (BCG) vaccination. Additionally, TST exhibits reduced sensitivity, especially among individuals with compromised immune systems, such as those living with HIV/AIDS [77,78].

T cell-based Interferon-Gamma Release Assays (IGRAs), which measure the production of IFN-γ by T cells in response to *Mtb*-specific antigens like Early Secretory Antigen Target-6 (ESAT-6) and Culture Filtrate Protein-10 (CFP-10), have gained popularity as TB diagnostic tools.

IGRAs offer several advantages over TST, including increased specificity and reduced cross-reactivity with BCG vaccination and most nontuberculous mycobacteria. They are also less influenced by prior *Mtb* exposure, making them particularly useful in BCG-vaccinated populations. However, despite these advantages, challenges persist regarding their inability to accurately distinguish between different stages within the *Mtb* infection spectrum and predict disease progression. Of note, the inability of IGRAs to reliably differentiate between active TB disease and LTBI as well as to predict active disease is a significant drawback and limits their use, especially in high-burden settings [[79], [80], [81]].

Previous studies that evaluated the immunogenicity of Rpfs in individuals who were exposed to active TB patients (mainly household contacts) indicated that stimulation of whole blood cells with Rpfs elicited the production of high amounts of IFN-γ (and other biomarkers) in both overnight (primarily, effector T cell) and long term (potentially a mixture of effector and central memory responses) culture assays [62,71,72,82].

Rpfs could potentially distinguish between infected individuals and those with active TB disease in both children and adults [66,83]. *In vitro* studies have revealed that IFN-γ-producing CD4^+^ T-cells specific to Rpfs (A, B, D, and E) are less abundant in patients with active TB compared to individuals with TB infection [35,64,67,68,84]. In addition, elevated IFN-γ responses to Rpfs might indicate an increased likelihood of progressing to active TB disease shortly following initial infection [71]. Table 1 summarizes previous studies that evaluated the diagnostic value IFN-γ in response to Rpfs.

**Table 1:** Abilities of the IFN-γ in response to Rpf antigens to discriminate between different groups.

**Study**	**Country**	**Year**	**Subjects**	**Rpf**	**PTB**	**LTBI**		**HCs**			***p-*value**	**AUC**			**Cut-off (pg/mL)**	Sensitivity (%)		**Specificity (%)**	
					**IFN-γ (pg/ml), median (IQR)**														
Kassa et al. [70]	Ethiopia	2006	6 PTBs	A	223.6 ± 234.6 ◊	-		-			-	-	-			66.7		-	
				B	154.0 ± 152.1◊	-		-			-	-	-			50		-	
				C	126.1 ± 128 ◊	-		-			-	-	-			66.7		-	
				D	182.4 ± 307.8◊	-		-			-	-	-			33.3		-	
Chegou et al. [72]	South Africa	2006- 2007	23 PTBs, 101 HHCs	A	0 (0–0)	52.2 (15.2–137.7)		-			0.0007	0.8	< 1.2			78.3		85	
				B	0 (0–0)	27.2 (1–103.2)		-			0.001	0.79	< 0.3			78.3		80	
				C	0 (0–0)	4.0 (0–58.2)		-			0.012	0.72	< 1.3			87		60	
				D	0 (0–0)	25.8 (2.7–98.1)			-		0.002	0.78	< 0.9			78.3		80	
				E	0 (0–20.2)	29.2 (11.9–127.4)			-		0.003	0.76	< 10.3			73.9		80	
Arroyo et al. [35]	Colombia		21 PTBs, 20 HHCs	A	121.8 (19.3–437.2)	576.8 (181–1224)		-			0.009	0.74	454.9			65		81	
				D	15.5 (6.3–200.3)	349.5 (74.72–932.6)			-		0.013	0.73	28.36			85		61.9	
van Loon et al. [71]	Gambia	2017	93 HHCs*	A	-	34.6 (5.2–73)	3.0 (0.0–11.1)			0.03		-		-			-		-
				B	-	77.4 (21.3–235.7)		13.6 (0.0–31.6)			0.007	-		33.9			73		92
				C	-	45.0 (25.9–115.8)		24.9 (0.0–30.4)			0.03	-		-			-		-
				D	-	138.0 (67.5–340.5)		49.3 (16.3–105.1)			0.004	-		67			77		72
				E⁎⁎⁎															
				A	9.1 (0.0–75.3)	47.1 (7.1–129.8)		-			0.02	-		-			-		-
				B	-	-		-			>0.05	-		-			-		-
				C	-	-		-			>0.05	-		-			-		-
				D	60.1 (11.0–114.2)	91.1 (40.9–284.5)		-			0.03	-		-			-		-
				E	-	-		-			>0.05	-		-			-		-
Mao et al. [69]	China	2020- 2021	17 HHCs,20 PTBs, 25 LTBIs, 23 RTBs	A	-	-		-			0.834●	0.52		> 4331⸭			100		46.51
				B	-	-		-			0.717●	0.53		> 2250⸭			100		46.51
				C	-	-		-			0.589●	0.54		> 2925⸭			100		46.51
				D	-	-		-			0.804●	0.52		> 2644⸭			100		46.51
				E	-	-		-			0.679●	0.53		> 4037⸭			100		46.51
				A	-	-		-			< 0.0001٭	0.91		> 45,103⸭			69.57		96
				B	-	-		-			< 0.0001٭	0.89		> 33,699⸭			73.91		96
				C	-	-		-			< 0.0001٭	0.86		> 27,163⸭			69.57		96
				D	-	-		-			< 0.0001٭	0.9		> 38,003⸭			65.22		96
				E	-	-		-			< 0.0001٭	0.88		> 34,054⸭			73.91		96
Riaño et al. [67]	Colombia		19 PTBs, 30 HHCs	A	-	-		-			-	-		-			40		-
				B	-	-		-			-	-		-			20		-
				C	-	-		-			-	-		-			20		-
				D	-	-		-			-	-		-			-		-
				E	-	-		-			-	-		-			20		-
Huang et al. [37]	China		12 PTB, 18 HHCs	A	-	-		-			< 0.01	-		-			-		-
				D	-	-		-			< 0.01	-		-			-		-
Sutherland et al. [65]	Gambia, Ethiopia, Malawi, Uganda, and South Africa		262 HIV-PTBs, 454 HIV- HCCs, 204 HIV- HCs, 77 HIV+ PTBs, 87 HIV+ HCCs and 163 HIV+ HCs	A	-	-		-			0.006⸸, 0.012⸹	-		-			-		-
				B	-	-		-			0.006⸸ <0.0033⸹	-		-			-		-
				E	-	-		-			0.004	-		-			-		-
Schuck et al. [64]	Germany		20 PTBs, 22 LTBIs	B	-	-		-			*P*<0.01	-		-			-		-
				C	-	-		-			> 0.05	-		-			-		-
				E	-	-		-			<0.05	-		-			-		-
Serra-Vidal et al. [63]	-	2010- 2013	5 PTBs, 6 LTBIs, 7 HCs	D	22.5 (5.0, 59.5)	135.8 (1.5, 610.5)		148.5 (12.0, 257.0)			0.046	-		-			-		-
Commandeur et al. [62]	Norway, Netherlands		9 PTBs, 22 LTBIs, 20 HCs #	A	-	-		-			> 0.05	-		-			-		-
				D	-	-		-			<0.05	-		-			-		-
Coppola et al. [61]	Spain		18 PTBs, 17 LTBIs ⁎⁎	A	-	47		-			> 0.05	-		-			-		-
				B	-	47		-			<0.05	-		-			-		-
				D	-	47		-			<0.05	-		-			-		-
				E	-	47		-			> 0.05	-		-			-		-

PTB: pulmonary tuberculosis, LTBIs: latent tuberculosis infections, HCs: healthy controls, AUC: area under the curve, Rpfs: resuscitation-promoting factors

◊ mean ± SD, ٭ distinguish RTBs from LTBIs

● distinguish LTBIs from TB-infected individuals

⁎ children aged below 15 years who were permanent household contacts of an adult smear-positive index TB

⁎⁎ 20 adults and 15 adolescents with PTB, ***not mentioned

⸸ HIV negative

⸹ HIV positive

⸭ mg/ml

# group of Dutch individuals, including 9 tuberculosis (TB) patients, 10 tuberculin skin test (TST)-positive individuals (indurations of ≥10 mm), 10 BCG-vaccinated individuals, and 10 non-BCG-vaccinated, TST-negative, healthy individuals, as well as 12 Norwegian TST-positive individuals.

Rpf antigens (RpfA and RpfD) have elicited higher IFN-γ production in stimulated Peripheral Blood Mononuclear Cells (PBMCs) from individuals with LTBI compared to those with active TB and exhibited a high probability of discriminating disease status (with area under the curve (AUC) > 0.70) [35]. Notably, these antigens were incorporated into predictive models for disease status, aligning with findings by Chegou and co-workers, who identified Rv0867c (RpfA) and Rv2389c (RpfD) as part of antigen combinations distinguishing between household contacts (HHC) and active TB [72].

These studies collectively suggest that Rpf-based IGRAs might address the shortcomings of current commercial IGRAs if developed. However, it's worth noting that the response to Rpf antigens can vary depending on the geographical location [13,65].

Since IGRAs primarily depend on T cell immune responses, their effectiveness in detecting TB infection in immunocompromised states, such as HIV co-infection where T cell dysfunction or exhaustion is common, may be suboptimal. Consequently, assessing the B cell response, specifically antibodies against mycobacterial Rpf proteins, in individuals co-infected with HIV and TB could offer valuable insights [74].

### 5.2 Rpfs as targets for vaccine development

The development of effective vaccines against TB remains a global priority in the fight against this persistent infectious disease, however, identifying the optimal antigens to incorporate into these vaccines has been challenging and remains incomplete [62]. Rpfs have emerged as promising targets for vaccine development, given their multifaceted role in TB infection and their ability to stimulate immune responses. Studies assessing the immune response to Rpf antigens, along with other latency-associated antigens, have shown encouraging results, indicating their potential as candidates for prophylactic vaccines targeting LTBI [13].

The demonstrated ability of latency-associated antigens, including Rpfs, DosR regulon-encoded antigens, and starvation regulon-encoded antigens, to stimulate cellular immune responses against LTBI underscores their potential as candidate antigens for immunization in prophylactic vaccines against the latent stage of TB infection [14,59,65,70,82,85].

Vaccination approaches employing Rpfs, particularly RpfB, and RpfE, have demonstrated varying degrees of efficacy in preclinical studies using mouse models. While RpfB vaccination elicited significant T cell responses, the level of protection against virulent *Mtb* challenge was moderate compared to conventional BCG vaccination [82]. Conversely, different vaccination approaches for RpfE have yielded mixed results. Vaccination with recombinant RpfE resulted in prolonged survival and reduced bacterial burden following *Mtb* exposure [86]. These contrasting outcomes suggest that the efficacy of Rpfs as vaccine candidates may vary depending on the specific antigen and vaccination approach employed [59].

While the RpfD pDNA vaccine resulted in a minor IFN-γ response, early findings from Romano et al.'s study propose more encouraging outcomes for RpfA and RpfC pDNA vaccines [82]. These initial observations highlight the variability in immune response elicited by different Rpf DNA vaccines and emphasize the importance of further investigation into their specific immunogenic properties. Understanding these differences is crucial for refining vaccine strategies and optimizing their effectiveness in combatting TB [13].

**Fig. 3:**
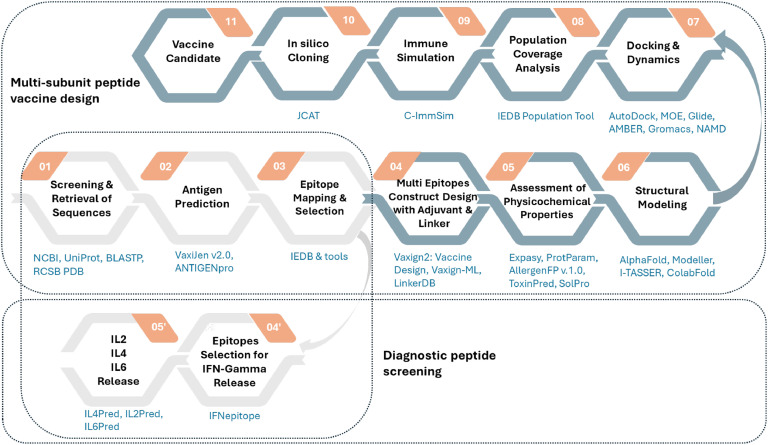
Schematic representation of computation workflow of (multi-epitope) vaccine design and development process in *Mtb* and diagnostic peptide screening.

In addition, computational vaccine design and development approaches against Rpfs in *Mtb* play a pivotal role in proposing promising vaccine candidates for further experimental research by facilitating analysis of the huge data sets, prioritization, design and conduct precise experiments [52,[87], [88], [89], [90]]. Briefly, computational studies in vaccine design and development help to identify potential antigenic targets, predict immune responses, and optimize vaccine candidates before moving to experimental phases. Even if computational approaches can vary based on the type of vaccine and the specific methodologies, the general workflow is presented herein (Fig. 3). Generally used computational tools and databases for each step were also tabulated in Table S2 in order to provide initial guidance for potential users. Note that some of the tools listed in Table S2 are parts of ELIXIR infrastructure (https://bio.tools/), IEDB: The Immune Epitope Database and Tools, SCRATCH Protein Predictor (https://scratch.proteomics.ics.uci.edu/), and ExPASy (https://www.expasy.org/). Taken together, since computational and experimental studies support and complement each other, both approaches should be considered for the development of effective vaccines against TB.

### 5.3 Drug targeting of Rpfs

Effectively targeting dormant bacilli, or persisters, is essential to completely eliminate the bacterial population in patients and reduce the treatment duration [[91], [92], [93]]. Therapeutic approaches targeting Rpfs offer promising strategies in the fight against TB by inhibiting the reactivation of dormant *Mtb* bacilli [14,94]. Small molecule inhibitors, like 2-nitrophenylthiocyanates (NPT), are designed to disrupt Rpf activity, aiming to halt the resuscitation process and subsequent reactivation of LTBI. Note that NPT is the first inhibitor group which was identified for the inhibition of the biological and enzymatic activity of the RpfB protein [12]. This approach is particularly relevant for individuals undergoing immunosuppressive treatments such as anti-TNFα inhibitors, where TB reactivation risk is heightened [11,12].

Among Rpfs, RpfB stands out as a compelling therapeutic target, opening new avenues for anti-TB drug development. Its identification presents opportunities to tackle drug-resistant strains and LTBI, critical challenges in TB management [95]. Notably, the lack of human homologs for RpfB ensures precise drug targeting, minimizing off-target effects [94,95]. Moreover, RpfB's extracellular nature facilitates easy access for small molecule inhibitors, bypassing the need for cellular entry to exert therapeutic effects [94]. These characteristics underscore RpfB's potential as a cornerstone in the development of highly effective and targeted anti-TB drugs, offering hope in overcoming TB's global burden. Ongoing research and drug development focusing on Rpfs hold promise for advancing TB treatment strategies and curbing TB's prevalence worldwide.

**Fig. 4:**
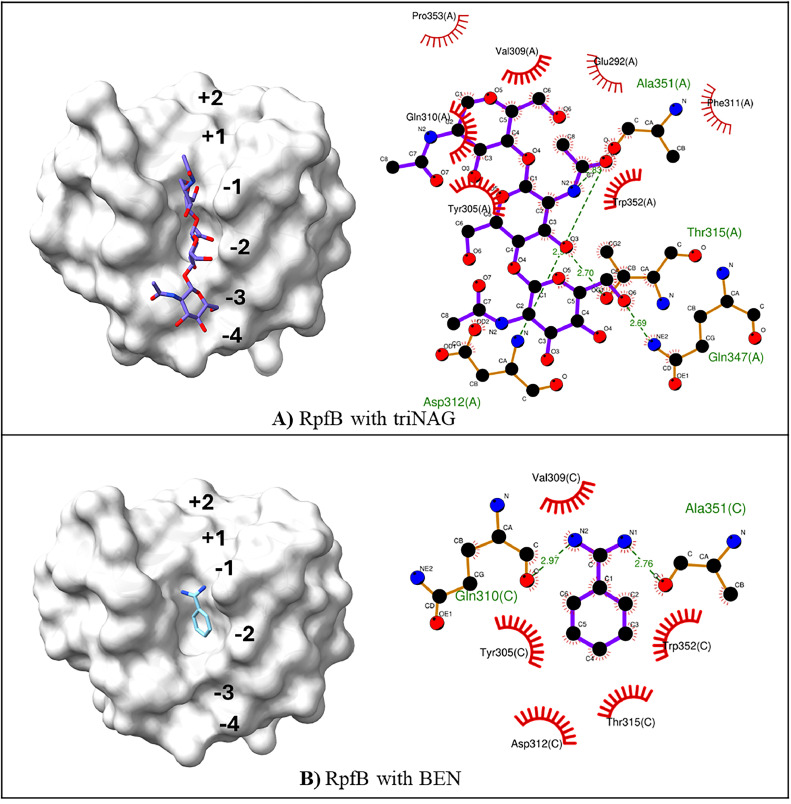
Surface representation of the catalytic site cleft of RpfB and accommodation of the (A) triacetyl-beta-chitotriose (triNAG, purple, PDB ID: 4KPM [44]) and (B) benzamidine ring (BEN, blue, PDB ID: 4EMN [45]) and 2D protein-ligand contacts in binding cleft by LigPlot+ using default settings [103].

Previous experimental and computational studies highlight the importance of identifying the Rpf catalytic domain, particularly binding cleft, and understanding its interaction patterns for designing potential inhibitors as therapeutic targets to prevent latent TB. In all Rpfs, the lysozyme-like catalytic domain is essential for its hydrolytic activity by breaking glycosidic bonds in the PGN, which is the main component of the *Mtb* cell wall. The catalytic domain includes the catalytic glutamic acid residues with adjacent hydrophobic pockets and disulfide bridges and these residues play a pivotal role in enzymatic activity/function and structural integrity (Fig. S1C). Structurally, six potential sugar binding sites are available in the catalytic cleft of RpfB defined as -4 to +2subsites [96]. Investigation of experimentally available complexes of RpfB shows that while triNAG binds to -3, -2, and -1, BEN binds to -2 (Fig. 4). Squeglia et al. reported H-bond interactions between the triNAG and the catalytic cleft (Glu292, Asp312, Gln310, Thr315, Ala351, and Gln347) (Fig. 4 A). Hydrophobic interactions (Pro353) and stacking interactions with Tyr305 were also observed. They also reported possible modulation of substrate access by the loop Gly348-Pro353 in RpfB which is close to the catalytic site [44]. Experimental and molecular dynamics (MD) findings indicate that the catalytic site becomes accessible when triNAG binds, due to the opening of this loop. More importantly, they suggest that RpfB breaks down the glycosidic bond of PGN between sites -1 and +1, involving Glu-292.

In inhibitor design, phytomolecules—bioactive compounds derived from plants—have gained popularity in modern therapeutics due to their minimal side effects, improved compliance, and effectiveness in eliminating organisms from the host [33]. Notably, several phytomolecules such as lipids/fatty acids, pure aromatics, phenolics, quinones, and alkaloids have been studied for their efficacy against non-replicating forms of *Mtb.* These studies highlight their potential as promising therapeutic agents in the fight against TB [97] In 2013, Ruggiero *et al*. investigated the interaction mechanism between the inhibitor 2–4-benzoyl-2-nitrophenyl thiocyanate (NPT7) and the RpfB and modifications of NPT7, revealing key molecular insights on inactivation mechanism (33). Critical residues for NPT7 binding to RpfB (Fig. S2) were detected and they highlighted the importance of thiocyanate moiety-mediated interactions, which establish hydrogen bonds with these residues. Substitution of the thiocyanate group with a hydroxyl group also completely abolished NPT7′s inhibitory activity, confirming the pivotal role of thiocyanate-mediated interactions. These findings suggest that modifications of the thiocyanate group binding to the catalytic site could enhance the affinity of the inhibitors to RpfB and conceivably lead to the development of more potent RpfB inhibitors. Even if limited number of *in silico* studies are available, inhibitor design studies targeting RbfB have started to receive more attention around 2020. In these studies, benzene-based inhibitors including phytomolecules [33,95,98,99] and antibiotics [58] were investigated for their ability to inhibit RbfB and identified promising candidates for further use by evaluating binding affinities, interactions, and complex stability. Fig. S2 provides information about interaction patterns between potential inhibitors and RpfB. In computational studies, virtual screening, drug design, ADME profiling, docking, molecular dynamics simulations (MD, REMD), analysis of RMSD, RMSF, interactions, binding free energy (MM-GBSA or MM-PBSA), and principal component analysis (PCA) were used to thoroughly investigate and validate the potential inhibitors as a drug target. Moreover, experimental validation —through *in vitro* and *in vivo* tests—of the promising RpfB inhibitors in *Mtb* identified through computational studies needs to be further studied to evaluate their safety and efficacy in treating TB infection. Furthermore, structure-activity relationship (SAR) studies are recommended to improve the potency and selectivity of these compounds. Exploring new natural compound libraries and under-researched medicinal plants may bring in new candidates with enhanced anti-TB properties. Lastly, the importance of combined experimental and computational efforts needs to be highlighted to fight TB in the latent stage.

## 6 Future research perspectives

Future research should delve deeper into understanding the dynamics of immune responses triggered by Rpfs across different stages of TB infection. This includes investigating the interplay between cellular and humoral immunity in response to Rpfs and exploring how these responses evolve from TB infection to active disease. Continued research is crucial to optimize Rpfs as vaccine candidates against TB. This includes evaluating the efficacy of different Rpfs individually and in combination with other antigens in animal models and translating these findings into clinical trials. Further exploration into small molecule inhibitors targeting Rpfs, particularly RpfB, is warranted. Future studies should aim to optimize inhibitor specificity, pharmacokinetics, and efficacy in preclinical and clinical settings. Investigating combination therapies involving Rpfs inhibitors with existing TB treatments could provide synergistic effects against drug-resistant TB strains and TB infection. Furthermore, understanding the distinct roles and regulations of each Rpf could provide insights into new strategies for TB control.

Design and development of Rpfs inhibitors could prevent the reactivation of LTBI, thereby reducing the risk of disease progression and transmission. Structural studies of Rpfs can aid in the design of such inhibitors by providing detailed insights into the active site and substrate interactions. When designing or evaluating inhibitors, it's crucial to consider non-covalent interactions (NCIs), namely hydrogen bonds, hydrophobic interactions, polar interactions, and electrostatic interactions between the binding cleft and inhibitors (triNAG and BEN as references). Additionally, since the catalytic site of Rpfs resemble each other, the potential inhibitors can be designed for them by using RpfB complexes as a reference. Overall, integrating experimental and computational studies enhances the design and development of inhibitors and vaccines.

## 7 Conclusion

In conclusion, Rpfs represent promising targets in the fight against TB, offering multifaceted roles in diagnosis, vaccine development, and drug targeting. Their ability to resuscitate dormant *Mtb* bacilli highlights their critical role in TB pathogenesis and persistence. Moving forward, it is crucial to unify efforts across basic research, translational studies, and clinical applications. By addressing current knowledge gaps and comprehending the diverse functionalities of Rpfs, we can advance TB control efforts and ultimately aim to eradicate this global health threat.

## CRediT authorship contribution statement

**Gamze Tanriver:** Writing – original draft, Visualization, Validation, Software, Methodology, Investigation. **Salman Ali Khan:** Writing – review & editing, Visualization, Validation, Investigation. **Artur Góra:** Writing – review & editing, Validation, Supervision, Software, Methodology, Conceptualization. **Novel N Chegou:** Writing – review & editing, Validation. **Shima Mahmoudi:** Writing – review & editing, Writing – original draft, Visualization, Supervision, Project administration, Methodology, Investigation, Funding acquisition, Conceptualization.

## Declaration of competing interest

The authors declare that they have no known competing financial interests or personal relationships that could have appeared to influence the work reported in this paper.

## Data Availability

Data will be made available on request.

## Appendix Supplementary materials

Image, application 1

Image, application 2
