# Supplementary material for: Exploring the multifaceted roles of resuscitation-promoting factors in tuberculosis: Implications for diagnosis, vaccine development, and drug targeting
Source: Biotechnol Rep (Amst). 2025 Mar 13;46:e00886. doi: 10.1016/j.btre.2025.e00886 (PMC11978375; doi:10.1016/j.btre.2025.e00886)
Supplement: Supplementary file 1 [file mmc1.docx]

| A  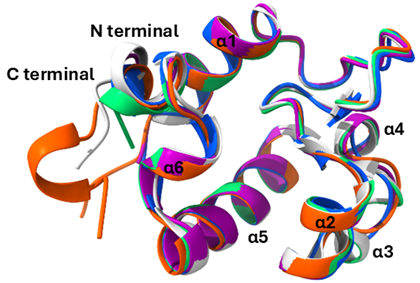 | B  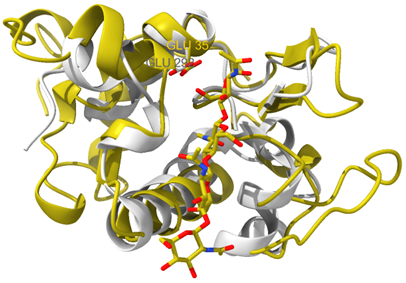 |
| --- | --- |
| C   \| RpfA \| RpfB \| RpfC \| RpfD \| RpfE \| \| --- \| --- \| --- \| --- \| --- \| \| catalytic Glu51 \| catalytic Glu292 \| catalytic Glu13 \| catalytic Glu61 \| catalytic Glu108 \| \| Val47, Trp56, Leu68, Phe70, Ile96, Trp111 \| Ile288, Trp297, Val309, Phe311, Ile337, Trp352 \| Val9, Trp18, Leu30, Phe32, Ile55, and Trp70 \| Ile57, Trp66, Leu78, Ile80, Ile103, Trp118 \| Ile104, Trp113,  Leu125, Phe127,  Ile150, Trp165 \| \| Cys50-Cys114 \| Cys291-Cys355 \| Cys12-Cys73 \| Cys60-Cys124 \| Cys15-Cys76 \| \| Tyr64, Thr71 \| Tyr305, Asp312 \| Lys26, Lys33 \| Leu74, Ser81 \| Leu121, Thr128 \| \| Gly109 \| Gly350 \| Asp68 \| Gly116 \| Arg 163 \| \| Gln69 \| Gln310 \| Gln31 \| Gln79 \| Arg126 \| \| Val113 \| Val354 \| Thr72 \| Lys120 \| Val167 \| \| His78 \| Asn319 \| Phe40 \| Gly89 \| Asn135 \| | |

Figure S1. Superimposed structures of catalytic domains of A) Rpf family B) RpfB and c-type lysozyme with tetra-NAG complex (PDB entry 1LMQ^1^) showing catalytic glutamates. RpfA in green, RpfB in light, RpfC in orange, RpfD in magenta, RpfE in blue, and c-type lysozyme with tetra-NAG complex in gold. C) Key conserved (yellow highlighted rows) and variant residues in the catalytic cleft of Rpfs. The figures were generated using ChimeraX (version 1.8).


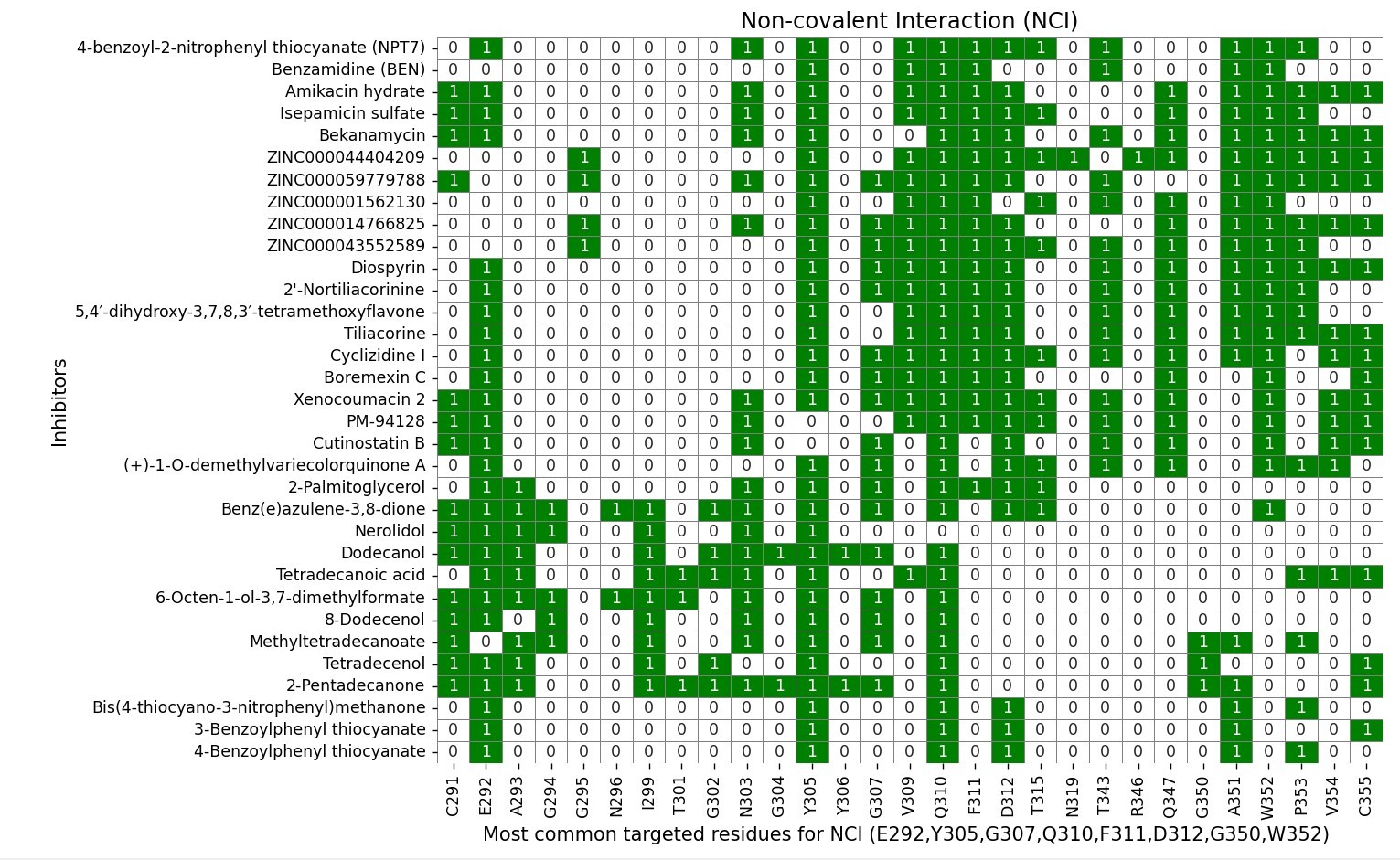


Figure S2. Summary of the Non-Covalent Interactions (NCI) between inhibitors and key amino acid residues in the catalytic domain of RpfB protein. Non-Covalent Interactions (NCI) indicated (1) or no Non-Covalent Interactions (NCI) (0). This figure was generated using in-house python script. ^2–8^

(1) Karlsen, S.; Hough, E. Crystal Structures of Three Complexes between Chito-Oligosaccharides and Lysozyme from the Rainbow Trout. How Distorted Is the NAG Sugar in Site D? urn:issn:0907-4449 1995, 51 (6), 962–978. https://doi.org/10.1107/S0907444995005105.

(2) Ruggiero, A.; Marchant, J.; Squeglia, F.; Makarov, V.; De Simone, A.; Berisio, R. Molecular Determinants of Inactivation of the Resuscitation Promoting Factor B from Mycobacterium Tuberculosis. J Biomol Struct Dyn 2013, 31 (2), 195–205. https://doi.org/10.1080/07391102.2012.698243.

(3) Dwivedi, V. D.; Arya, A.; Sharma, T.; Sharma, S.; Patil, S. A.; Gupta, V. K. Computational Investigation of Phytomolecules as Resuscitation-Promoting Factor B (RpfB) Inhibitors for Clinical Suppression of Mycobacterium Tuberculosis Dormancy Reactivation. Infection, Genetics and Evolution 2020, 83, 104356. https://doi.org/10.1016/j.meegid.2020.104356.

(4) Shah, M.; Khan, F.; Ahmad, I.; Deng, C. L.; Perveen, A.; Iqbal, A.; Nishan, U.; Zaman, A.; Ullah, R.; Ali, E. A.; Chen, K. Computer-Aided Identification of Mycobacterium Tuberculosis Resuscitation-Promoting Factor B (RpfB) Inhibitors from Gymnema Sylvestre Natural Products. Front Pharmacol 2023, 14, 1325227. https://doi.org/10.3389/FPHAR.2023.1325227/BIBTEX.

(5) Chouhan, M.; Tiwari, P. K.; Moustafa, M.; Chaubey, K. K.; Gupta, A.; Kumar, R.; Sahoo, A. K.; Azhar, E. I.; Dwivedi, V. D.; Kumar, S. Inhibition of Mycobacterium Tuberculosis Resuscitation-Promoting Factor B (RpfB) by Microbially Derived Natural Compounds: A Computational Study. J Biomol Struct Dyn 2024, 42 (2), 948–959. https://doi.org/10.1080/07391102.2023.2208214.

(6) Kumar, G. S.; Dubey, A.; Panda, S. P.; Alawi, M. M.; Sindi, A. A.; Azhar, E. I.; Dwivedi, V. D.; Agrawal, S. Repurposing of Antibacterial Compounds for Suppression of Mycobacterium Tuberculosis Dormancy Reactivation by Targeting Resuscitation-Promoting Factors B. J Biomol Struct Dyn 2024, 42 (13), 6850–6862. https://doi.org/10.1080/07391102.2023.2245059.

(7) Rabaan, A. A.; Garout, M.; Aljeldah, M.; Al Shammari, B. R.; Alawfi, A.; Alshengeti, A.; Najim, M. A.; Alrouji, M.; Almuhanna, Y.; Alissa, M.; Mashraqi, M. M.; Alwashmi, A. S. S.; Alhajri, M.; Alateah, S. M.; Farahat, R. A.; Mohapatra, R. K. Anti-Tubercular Activity Evaluation of Natural Compounds by Targeting Mycobacterium Tuberculosis Resuscitation Promoting Factor B Inhibition: An in Silico Study. Mol Divers 2024, 28 (3), 1057–1072. https://doi.org/10.1007/s11030-023-10632-8.

(8) Demina, G. R.; Nikitushkin, V. D.; Shleeva, M. O.; Riabova, O. B.; Lepioshkin, A. Y.; Makarov, V. A.; Kaprelyants, A. S. Benzoylphenyl Thiocyanates Are New, Effective Inhibitors of the Mycobacterial Resuscitation Promoting Factor B Protein. Ann Clin Microbiol Antimicrob 2017, 16 (1). https://doi.org/10.1186/s12941-017-0244-7.
