# Supplementary material for: Exploring the multifaceted roles of resuscitation-promoting factors in tuberculosis: Implications for diagnosis, vaccine development, and drug targeting
Source: Biotechnol Rep (Amst). 2025 Mar 13;46:e00886. doi: 10.1016/j.btre.2025.e00886 (PMC11978375; doi:10.1016/j.btre.2025.e00886)
Supplement: Supplementary file 2 [file mmc2.docx]

Table S1. The members of the Rpf family of proteins in *M. tuberculosis*.

| Members | PDB | Type | Resolution | Apo/Complex | Length (aa) | Domain |
| --- | --- | --- | --- | --- | --- | --- |
| RpfA  (Rv0867c) | No structure available | | | | |  |
| RpfB (Rv1009) | 3EO5^1^ | X-ray | 1.83 Å | Apo | 171 | Catalytic, G5 |
|  | 1XSF^2^ | NMR |  | Apo | 108 | Catalytic |
|  | 5E27^3^ | X-ray | 2.60 Å | Apo | 248 | DUF348(DUF3), G5, Catalytic |
|  | 4KL7^4^ | X-ray | 1.45 Å | Apo | 80 | Catalytic |
|  | 4KPM^4^ | X-ray | 1.33 Å | Complex | 80 | Catalytic |
|  | 4EMN^5^ | X-ray | 1.17 Å | Complex | 81 | Catalytic |
| RpfC  (Rv1884c) | 4OW1^6^ | X-ray | 1.90 Å | Complex | 92 | Catalytic |
|  | 2N5Z^7^ | NMR |  | Apo | 82 | Catalytic |
| RpfD  (Rv2389c) | No structure available | | | | |  |
| RpfE  (Rv2450c) | 4CGE^8^ | X-ray | 2.76 Å | Apo | 75 | Catalytic |

Table S2. List of commonly used computational tools and databases for vaccine design and development.

| Category | | | Tool | | Description | | Website Link | |
| --- | --- | --- | --- | --- | --- | --- | --- | --- |
| Screening & Retrieval of Sequences | | | NCBI | | Sequence retrieval and analysis | | <https://www.ncbi.nlm.nih.gov/> | |
|  |  |  | UniProt | | Protein sequence and functional information | | <https://www.uniprot.org/> | |
|  |  |  | InterPro | | Protein family classification and domain prediction | | https://www.ebi.ac.uk/interpro/ | |
|  |  |  | Protein Variability Server | | Sequence variability analysis | | http://imed.med.ucm.es/PVS/ | |
|  |  |  | BLASTP | | Protein sequence alignment | | <https://blast.ncbi.nlm.nih.gov/Blast.cgi?PAGE=Proteins> | |
|  |  |  | RCSB PDB | | 3D structural data of biological molecules | | <https://www.rcsb.org/> | |
|  |  |  | Clustal Omega | | Multiple sequence alignment | | <https://www.ebi.ac.uk/jdispatcher/msa/clustalo> | |
|  |  |  | MAFFT | | Multiple sequence alignment software | | https://mafft.cbrc.jp/alignment/server/index.html | |
| Antigen Prediction | | | VaxiJen v2.0 | | Predicts antigenicity based on amino acid sequences. | | <https://www.ddg-pharmfac.net/vaxijen/VaxiJen/VaxiJen.html> | |
|  |  |  | ANTIGENpro | | Machine-learning-based tool for antigenicity prediction. | | https://scratch.proteomics.ics.uci.edu/ | |
| Epitope Mapping and Selection in Vaccine Design | | | IEDB | | Predicts B-cell and T-cell epitopes. | | <https://www.iedb.org/>  <https://nextgen-tools.iedb.org/> | |
|  |  |  | BepiPred 3.0 | | Predicts linear B-cell epitopes. | | <https://services.healthtech.dtu.dk/services/BepiPred-3.0/> | |
|  |  |  | DiscoTope 2.0 IEDB | | Predicts discontinuous B-cell epitopes. | | <http://tools.iedb.org/discotope/> | |
|  |  |  | ElliPro IEDB | | Identifies linear and discontinuous epitopes. | | <http://tools.iedb.org/ellipro/> | |
|  |  |  | NetMHCpan4.1 | | Predicts binding affinities of peptides to MHC class I molecules. | | https://services.healthtech.dtu.dk/services/NetMHCpan-4.1/ | |
|  |  |  | NetMHCIIpan4.0 | | Predicts binding affinities of peptides to MHC class II molecules. | | https://services.healthtech.dtu.dk/services/NetMHCIIpan-4.0/1-Submission.php | |
|  |  |  | SVMTriP | | Predicts T-cell epitopes using support vector machines. | | <http://sysbio.unl.edu/SVMTriP/prediction.php> | |
|  |  |  | ABCPred | | Predicts B-cell epitopes using artificial neural networks. | | <https://webs.iiitd.edu.in/raghava/abcpred/> | |
|  |  |  | MHC-I Binding Predictions | | Tool for predicting MHC class I binding affinity of peptides. | | <http://tools.iedb.org/mhci/> | |
|  |  |  | MHC-II Binding Predictions | | Tool for predicting MHC class II binding affinity of peptides. | | <http://tools.iedb.org/mhcii/> | |
| Adjuvant and Linker Design | | | JCAT (Java Codon Adaptation Tool) | | Codon optimization tool for vaccine constructs. | | <http://www.jcat.de/> | |
|  |  |  | LinkerDB | | Database for searching and visualising protein inter-domain linkers | | <https://www.ibi.vu.nl/programs/linkerdbwww/> | |
| Vaccine Construct Design | | | Vaxign2: Vaccine Design | | vaccine target prediction and analysis system based on the principle of reverse vaccinology. | | <https://violinet.org/vaxign2> | |
|  |  |  | Vaxign-ML (Vaxign - Machine Learning) | | Machine learning-based vaccine candidate prediction and analysis system based on the principle of reverse vaccinology. | | <https://violinet.org/vaxign/vaxign-ml/> | |
|  |  |  | Disulfide by Design 2 | | Web-based tool for disulfide engineering in proteins | | <http://cptweb.cpt.wayne.edu/DbD2/index.php> | |
| Assessment of Physicochemical Properties | Allergenicity and Safety | AllergenFP v.1.0 | | Allergenicity prediction tool based on fingerprint-based approach. | | <https://ddg-pharmfac.net/AllergenFP/> | |  |
|  |  | AllerTop v.2.0 | | Machine learning-based allergenicity prediction tool. | | https://www.ddg-pharmfac.net/AllerTOP/ | |  |
|  |  | AlgPred | | Tool for predicting allergenic proteins and peptides. | | https://webs.iiitd.edu.in/raghava/algpred/submission.html | |  |
|  | Toxicity Prediction | ToxinPred | | Predicts toxicity of peptides and proteins. | | http://crdd.osdd.net/raghava/toxinpred/ | |  |
|  | Immunogenicity Prediction | Class I Immunogenicity | | Tool for predicting MHC class I immunogenicity. | | http://tools.iedb.org/immunogenicity/ | |  |
|  |  | CD4 T Cell Immunogenicity Prediction | | Predicts CD4 T cell epitope immunogenicity. | | http://tools.iedb.org/CD4episcore/ | |  |
|  | Solubility Prediction | SolPro | | Predicts protein solubility upon overexpression. | | <https://scratch.proteomics.ics.uci.edu/> | |  |
|  |  | Protein-Sol | | solubility prediction calculations | | <https://protein-sol.manchester.ac.uk/> | |  |
|  | Physicochemical Analysis | ProtParam | | Calculates physical and chemical parameters of proteins. | | <https://web.expasy.org/protparam/>  <https://protparam.net/index.html> | |  |
|  |  | SwissADME | | Calculates physicochemical descriptors and predict ADME parameters, pharmacokinetic properties, so on. | | http://www.swissadme.ch/ | |  |
| Structural Modeling | | | AlphaFold | | AI-based protein structure prediction. | | https://alphafold.ebi.ac.uk/  https://alphafoldserver.com/about | |
|  |  |  | SWISS-MODEL | | Homology-based protein modeling tool. | | https://swissmodel.expasy.org/ | |
|  |  |  | I-TASSER | | Iterative Threading ASSEmbly Refinement; a tool for protein structure prediction. | | https://zhanggroup.org/I-TASSER/ | |
|  |  |  | MODELLER | | Homology modeling program. | | <https://salilab.org/modeller/> | |
|  |  |  | ColabFold | | Fast and accessible implementation of AlphaFold2. | | <https://colab.research.google.com/github/sokrypton/ColabFold/blob/main/AlphaFold2.ipynb> | |
| Docking & Dynamics | | | AutoDock | | Molecular docking software for predicting how small molecules, like ligands, bind to a receptor. | | <https://autodock.scripps.edu/> | |
|  |  |  | AutoDock Vina | | Enhanced version of AutoDock with improved speed and accuracy. | | <https://vina.scripps.edu/> | |
|  |  |  | MOE (Molecular Operating Environment) | | Comprehensive tool for molecular modeling, docking, and simulations. | | <https://www.chemcomp.com/en/Products.htm> | |
|  |  |  | Glide | | High-throughput docking tool with precision levels from HTVS to XP. | | <https://www.schrodinger.com/platform/products/glide/> | |
|  |  |  | SwissDock | | Web-based docking tool using the EADock algorithm. | | <https://www.swissdock.ch/> | |
|  |  |  | AMBER | | Conducts molecular dynamics simulations and analysis. | | <https://ambermd.org/index.php> | |
|  |  |  | GROMACS | | Conducts molecular dynamics simulations and analysis. | | <https://www.gromacs.org/> | |
|  |  |  | NAMD | | Advanced molecular dynamics tool and analysis. | | <https://www.ks.uiuc.edu/Research/namd/> | |
| Population Coverage Analysis | | | IEDB Population Tool | | Estimates population coverage of T-cell epitopes. | | <http://tools.iedb.org/population/> | |
| Immune Simulation | | | C-ImmSim | | Models immune response to vaccine constructs. (C-language version of IMMSIM) | | <https://kraken.iac.rm.cnr.it/C-IMMSIM/index.php> | |
| In silico cloning | | | JCAT (Java Codon Adaptation Tool) | | a novel tool to adapt codon usage of a target gene to its potential expression host | | <http://www.jcat.de/> | |
| IFN-gamma inducing peptides | | | IFNepitope(Interferon-gamma epitope) | | web server is developed for users working in the field of vaccine design, predict and design IFN-gamma inducing peptides | | <http://crdd.osdd.net/raghava/ifnepitope/> | |
| Interleukins inducing peptides Prediction | | | IL2Pred  IL4Pred  IL6Pred | | web server developed to predict interleukins inducing peptides | | <https://webs.iiitd.edu.in/raghava/il2pred/stat.php>  <https://webs.iiitd.edu.in/raghava/il6pred/>  <http://crdd.osdd.net/raghava/il4pred/> | |

References

(1) Ruggiero, A.; Tizzano, B.; Pedone, E.; Pedone, C.; Wilmanns, M.; Berisio, R. Crystal Structure of the Resuscitation-Promoting Factor ΔDUFRpfB from M. Tuberculosis. J Mol Biol 2009, 385 (1), 153–162. https://doi.org/10.1016/J.JMB.2008.10.042.

(2) Cohen-Gonsaud, M.; Barthe, P.; Bagnéris, C.; Henderson, B.; Ward, J.; Roumestand, C.; Keep, N. H. The Structure of a Resuscitation-Promoting Factor Domain from Mycobacterium Tuberculosis Shows Homology to Lysozymes. Nat Struct Mol Biol 2005, 12 (3), 270–273. https://doi.org/10.1038/nsmb905.

(3) Ruggiero, A.; Squeglia, F.; Romano, M.; Vitagliano, L.; De Simone, A.; Berisio, R. The Structure of Resuscitation Promoting Factor B from M. Tuberculosis Reveals Unexpected Ubiquitin-like Domains. Biochimica et Biophysica Acta (BBA) - General Subjects 2016, 1860 (2), 445–451. https://doi.org/10.1016/J.BBAGEN.2015.11.001.

(4) Squeglia, F.; Romano, M.; Ruggiero, A.; Vitagliano, L.; De Simone, A.; Berisio, R. Carbohydrate Recognition by RpfB from Mycobacterium Tuberculosis Unveiled by Crystallographic and Molecular Dynamics Analyses. Biophys J 2013, 104 (11), 2530–2539. https://doi.org/10.1016/j.bpj.2013.04.040.

(5) Ruggiero, A.; Marchant, J.; Squeglia, F.; Makarov, V.; De Simone, A.; Berisio, R. Molecular Determinants of Inactivation of the Resuscitation Promoting Factor B from Mycobacterium Tuberculosis. J Biomol Struct Dyn 2013, 31 (2), 195–205. https://doi.org/10.1080/07391102.2012.698243.

(6) Chauviac, F.-X.; Robertson, G.; Quay, D. H. X.; Bagnéris, C.; Dumas, C.; Henderson, B.; Ward, J.; Keep, N. H.; Cohen-Gonsaud, M. The RpfC (Rv1884) Atomic Structure Shows High Structural Conservation within the Resuscitation-Promoting Factor Catalytic Domain. Acta Crystallogr F Struct Biol Commun 2014, 70 (8), 1022–1026. https://doi.org/10.1107/S2053230X1401317X.

(7) Maione, V.; Ruggiero, A.; Russo, L.; De Simone, A.; Pedone, P. V.; Malgieri, G.; Berisio, R.; Isernia, C. NMR Structure and Dynamics of the Resuscitation Promoting Factor RpfC Catalytic Domain. PLoS One 2015, 10 (11), e0142807. https://doi.org/10.1371/JOURNAL.PONE.0142807.

(8) Mavrici, D.; Prigozhin, D. M.; Alber, T. Mycobacterium Tuberculosis RpfE Crystal Structure Reveals a Positively Charged Catalytic Cleft. Protein Science 2014, 23 (4), 481–487. https://doi.org/10.1002/pro.2431.
